# Integrative analysis identifies STX16 as a prognostic and immune-associated biomarker in ccRCC

**DOI:** 10.1038/s41598-025-24921-9

**Published:** 2025-11-20

**Authors:** Yumeng Chai, Runze Liu, Yuanshan Cui, Zhongbao Zhou, Yong Zhang

**Affiliations:** 1 Department of Urology, Beijing Tiantan Hospital, Capital Medical University, No.119 South 4th Ring West Road, Fengtai District, Beijing, 100070 China; 2 Department of Urology, Weifang People’s Hospital, No. 151 GuangwenStreet, Weifang, Shandong China

**Keywords:** Cancer genetics, Gene expression

## Abstract

STX16 has been studied in various cancers, where it is suggested to regulate tumor cell proliferation, migration, and invasion by affecting vesicle trafficking and signal transduction pathways. However, its specific role in clear cell renal cell carcinoma (ccRCC) remains unclear. This study aims to systematically investigate the expression patterns, clinical significance, prognostic value and functional role of STX16 in ccRCC. Bioinformatics analyses using TCGA, CTPAC, TIMER, and UALCAN databases evaluated STX16 expression, clinical correlations, prognosis, and immune infiltration. Functional enrichment, co-expression, and PPI analyses explored STX16-associated pathways. Single-cell sequencing elucidated tumor microenvironment heterogeneity. Laboratory validation included Western blot, immunohistochemistry, and functional assays with siRNA-mediated STX16 knockdown in ccRCC cell lines. STX16 was significantly upregulated at mRNA and protein levels in ccRCC tissues. High STX16 expression correlated with advanced tumor stages, poor overall survival (OS), and disease-specific survival (DSS). Multivariable Cox regression identified STX16 as an independent prognostic factor. STX16 influenced immune infiltration, particularly involving CD4 + T cells, macrophages, and neutrophils, and was associated with immune pathways. Single-cell sequencing revealed heterogeneous STX16 expression across tumor microenvironment cell types. STX16 knockdown inhibited proliferation, migration, and invasion of ccRCC cell lines. STX16 is upregulated in ccRCC and acts as an independent prognostic factor associated with poor outcomes. Its role in promoting tumor progression and modulating immune infiltration highlights STX16 as a potential biomarker and therapeutic target in ccRCC.

**Supplementary Information:**

The online version contains supplementary material available at 10.1038/s41598-025-24921-9.

## Introduction

Clear cell renal cell carcinoma (ccRCC) is the most common subtype of renal cell carcinoma (RCC), accounting for approximately 75–80% of all RCC cases^1^. The global burden of kidney cancer remains substantial, with approximately 434,419 new cases and 155,702 deaths estimated in 2022^2^. Despite advancements in early detection and treatment, the prognosis for ccRCC remains poor, especially in patients with advanced disease, where the five-year survival rate remains low^3^. The molecular pathogenesis of ccRCC is complex, involving multiple genetic mutations and altered signaling pathways. This has led to a growing interest in identifying novel molecular biomarkers and therapeutic targets^4^. With the advances of high-throughput sequencing technologies and bioinformatics tools, our understanding of ccRCC’s genomic landscape has expanded, offering new opportunities to identify potential therapeutic targets^5^.

Syntaxin 16 (STX16) is a membrane-bound protein belonging to the SNARE (soluble *N*-ethylmaleimide-sensitive factor attachment protein receptor) family, primarily involved in intracellular membrane trafficking and fusion^6^. Current research on STX16 primarily focuses on its role in pseudohypoparathyroidism (PHP), particularly PHP1b, in which STX16 deletions are implicated in abnormal methylation of the GNAS locus, leading to endocrine resistance. STX16 mutations are associated with various phenotypes, including parathyroid hormone resistance and early-onset obesity, highlighting its involvement in both metabolic and hormonal regulation^7,8^. However, its role in cancer remains largely unexplored. Preliminary studies suggest that STX16 may regulate tumor cell proliferation, migration, and invasion by affecting vesicle trafficking and signal transduction pathways^9^. In some cancer types, STX16 expression levels have been correlated with patient prognosis, but its specific role in ccRCC has not yet been thoroughly investigated^10,11^.

Given STX16’s potential involvement in intracellular transport and signaling pathways, it may play a crucial role in tumor progression. However, a systematic analysis of STX16 in ccRCC is lacking. A comprehensive bioinformatics analysis, combined with functional experiments, could provide novel insights into the role of STX16 in ccRCC and its potential as a diagnostic biomarker or therapeutic target. These findings could aid in developing personalized treatment strategies for ccRCC.

This study aims to systematically analyze the differential expression of STX16 in ccRCC using transcriptomic and proteomic data and investigate the potential biological functions of STX16. Additionally, we will evaluate the clinical significance of STX16 expression in relation to prognostic and diagnostic relevance. Finally, experimental validation and functional assays will be conducted to elucidate the potential biological role of STX16 in ccRCC progression.

## Materials and methods

### Data sources

**Fig. 1:**
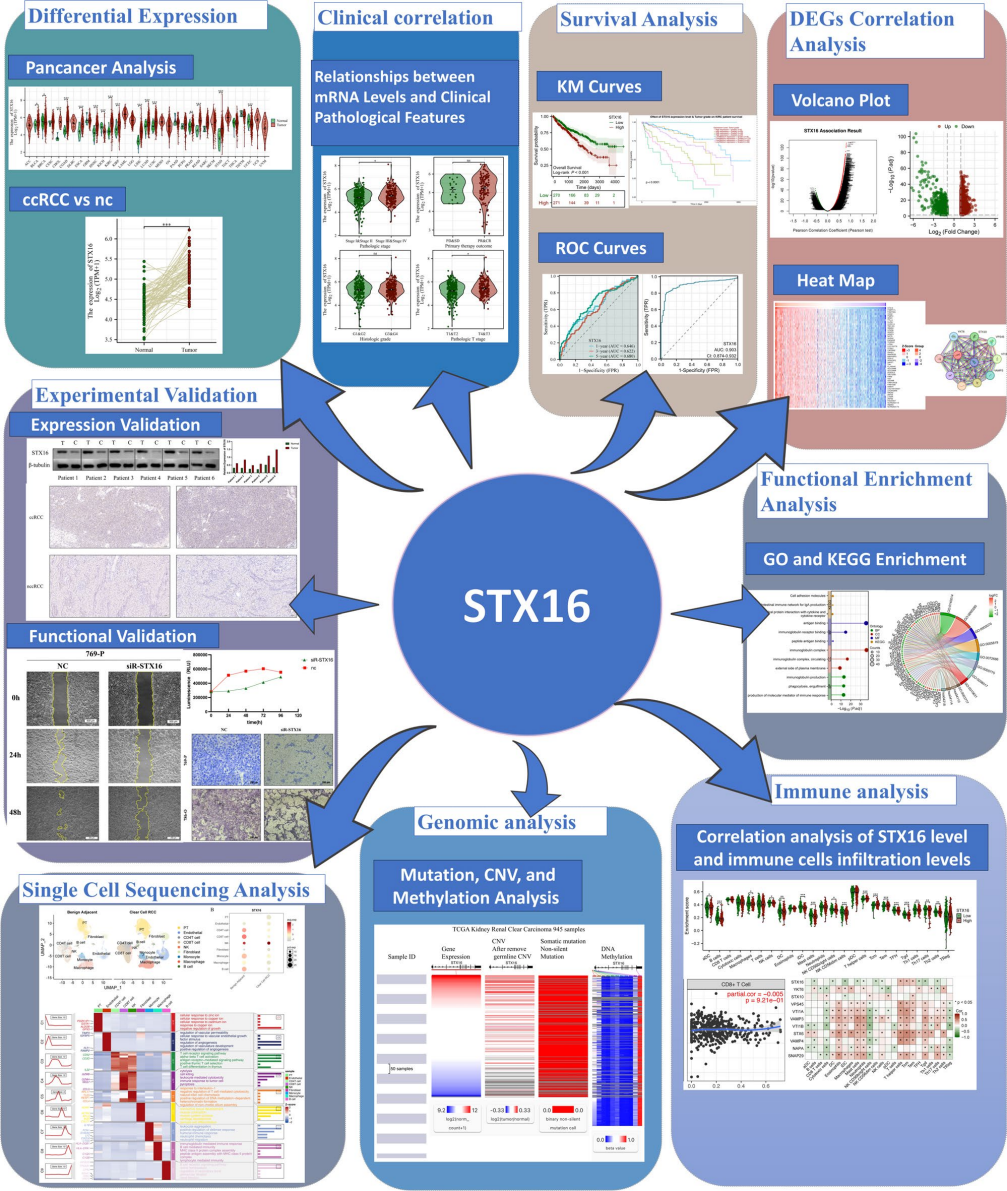
The workflow of this study.

Transcriptomic and clinical data were obtained from publicly accessible databases, such as The Cancer Genome Atlas (TCGA) and the Gene Expression Omnibus (GEO). Intergroup comparison and survival analysis were conducted within the samples including both neoplastic and non-neoplastic tissues. The workflow of this study is presented in Fig. 1.

### Differential expression and its correlation with clinical parameters

We used the stats and car R packages to analyze the difference in STX16 expression between tumor and normal tissues and used ggplot2 to visualize the results. The association between STX16 expression and clinical parameters was assessed using Spearman and Pearson correlation analyses, with significance set at *p* < 0.05.

### Univariable and multivariable cox regression analysis and survival analysis

Univariable and multivariable Cox regression analyses were conducted to determine whether STX16 expression and clinical parameters such as age, gender, TNM stage, pathological stage, race, histologic grade and serum calcium could independently predict patient outcomes. Furthermore, the impact of STX16 on prognosis was explored using Kaplan-Meier survival curves for overall survival. Diagnostic and time-dependent ROC curves were generated to assess the predictive accuracy of STX16. Analyses were performed using the survival and timeROC R packages.

### Differential expression analysis and visualization

Differential expression analysis was conducted to identify significant differentially expressed genes (DEGs) between ccRCC and adjacent normal tissue samples. DEGs were identified based on the criteria: |log2 fold change| >1 and adjusted P-value < 0.05. Volcano plots were generated to visualize the distribution of DEGs across the datasets.

For co-expression analysis, the expression profiles of DEGs were intersected across datasets using a Venn diagram, created with the VennDiagram R package. Genes present in the intersection were considered commonly expressed DEGs. Subsequently, a heatmap was generated using the heatmap R package to illustrate the expression patterns of these common DEGs.

### Protein–protein interaction (PPI) network

The PPI network of STX16-associated genes was constructed using the STRING database (https://cn.string-db.org/), with a minimum interaction score of 0.4 set as the threshold. The resulting network was visualized and analyzed to identify key nodes and their interactions.

### Functional enrichment analysis and visualization

Gene Ontology (GO) and Kyoto Encyclopedia of Genes and Genomes (KEGG) pathway enrichment were performed using the ClusterProfiler package in R^12–14^. Functional clustering was done to group genes by biological function, categorizing GO terms into molecular function (MF), biological process (BP), and cellular component (CC). We integrated enrichment analysis with logFC values of associated molecules to calculate Z-scores for each enriched term or pathway. A positive Z-score indicates pathway upregulation, while a negative Z-score suggests downregulation, helping us determine the direction of regulation for the biological processes involved.

To visualize the enrichment results, lollipop charts and network diagrams were constructed to display the top enriched terms for GO and KEGG analyses and highlight relationships among enriched terms. A bubble plot and a chord diagram illustrated combined analysis results of GO and KEGG enrichment analysis integrated with logFC. All visualizations were created using ggplot2 R packages.

### Immune infiltration

Based on the ssGSEA algorithm provided in the R package GSVA, immune infiltration was calculated using the markers of 24 immune cell types provided in the article by Bindea, Gabriela et al.^15,16^. We used TIMER2 (http://timer.cistrome.org/) and Kaplan-Meier plotter (http://kmplot.com/analysis/) to evaluate the tumor immune infiltration and survival analysis in different immune cell subpopulations, which was integrated and visualized through heatmap.

### Gene expression, copy number variation, somatic mutation and DNA methylation analysis

The UCSC Xena database (http://xena.ucsc.edu/), a comprehensive platform integrating approximately 200 public genomic databases such as TCGA, ICGC, and GTEx, were utilized to analyze copy number variation (CNV), somatic mutations, gene expression, and DNA methylation levels of STX16 in ccRCC.

### Single-cell sequencing analysis

Single-cell RNA sequencing (scRNA-seq) data was obtained from five GSE datasets: GSE222703, GSE156632, GSE207493, GSE242299 and GSE159115, downloaded from the GEO database (https://www.ncbi.nlm.nih.gov/geo/). ScRNA-seq data was analyzed using the Seurat package in R. After data processing and clustering, STX16 expression across different cell types in ccRCC and benign adjacent tissues was evaluated to understand its role at the cellular level.

### Patient samples

ccRCC and paired adjacent normal renal tissues were collected from six patients who underwent radical or partial nephrectomy in 2024 in the urology department of Beijing Tiantan Hospital. Tissue samples were immediately snap-frozen in liquid nitrogen following surgical excision. We collected the clinical and pathological characteristics from 104 patients, including information on gender, age, TNM stage, and pathological grade. This study was approved by the Ethics Committee of our hospital and the approval number is KY2024-406-01. All patients provided written informed consent prior to their participation. All methods were performed in accordance with the relevant guidelines and regulations.

### Western blot analysis

Cells were lysed in RIPA buffer (Servicebio, China) supplemented with 1% protease inhibitor and incubated for 30 min. After centrifugation, the protein supernatant was collected and mixed with 5× protein loading buffer (Coolaber, China). The mixture was then heated at 100 °C for 10 min to denature the proteins before loading onto SDS-PAGE gels for electrophoresis. Following separation, proteins were transferred onto a nitrocellulose (NC) membrane.

The membranes were blocked with TBST containing 5% skimmed milk for 2 h at room temperature and then incubated overnight at 4 °C with primary antibodies. The following day, secondary antibody incubation was performed at room temperature for 1 h. Protein bands were visualized using a Clinx Gel Documentation and Analysis system, and band intensity was quantified with ImageJ software.

The antibodies used in this Western blot included anti-STX16 (1:1000, Huabio, China), anti-β-tubulin (1:1000, Cell Signaling Technology, USA), goat anti-rabbit secondary antibody (1:5000, Cell Signaling Technology, USA), and goat anti-mouse secondary antibody (1:5000, Cell Signaling Technology, USA).

#### **Immunohistochemistry (IHC) analysis**

Formalin-fixed, paraffin-embedded tissue samples were sectioned into 3-µm thick slices and incubated at 57 °C for 90–120 min. The sections were then dewaxed in xylene, rehydrated through a graded ethanol series, and boiled in citrate buffer (0.01 M, pH 6.0) for 2 min for antigen retrieval. Endogenous peroxidase activity was quenched using 0.3% hydrogen peroxide.

The sections were incubated overnight at 4 °C with primary antibodies. After washing three times with PBS (0.01 M, 5 min each), they were incubated with a biotin-labeled secondary antibody at room temperature for 1 h. The immunoreactivity was visualized using 3,3’-diaminobenzidine tetrahydrochloride (DAB) as the chromogen, and hematoxylin was used for nuclear counterstaining.

The primary antibody used in IHC analysis was anti-STX16 (1:1000, Huabio, China).

### Cell lines and cell culture

The ccRCC cell lines (786-O and 769-P) and the human normal renal proximal convoluted tubular cell line (HK-2) were obtained from the Cell Bank of the Chinese Academy of Sciences. HK-2 cells were cultured in Dulbecco’s Modified Eagle Medium (DMEM, Biological Industries, Israel), while all ccRCC cell lines were cultured in RPMI 1640 medium (Biological Industries, Israel). Both media were supplemented with 10% fetal bovine serum (FBS) and 1% penicillin-streptomycin. All cell lines were incubated at 37 °C in a humidified atmosphere containing 5% CO_2_.

### Transfection

Small interfering RNAs targeting STX16 (siR-STX16) and their corresponding negative control (NC) were synthesized by SyngenTech (China). Transfections were performed using Lipofectamine 3000 reagent (Invitrogen, USA) according to the manufacturer’s instructions. After transfection, cells were incubated for 24–48 h before further experiments. The sequences of the siRNAs and primers used in the study are detailed in Table 1 and the efficiency of the three designed primers was validated using qRT-PCR, as shown in the supplementary figure. The transfection efficiency was validated by assessing the protein expression levels of STX16 using WB analysis.

**Table 1:** The sequences of the siRNAs and primers.

No.	Name	Sequence (5′→3′)
1	STX16 (human) siRNA-440	GGAACAUGCCAUUGAGAUATT
2	STX16 (human) siRNA-644	CAUGAAGAAUCGAGAGGAATT
3	STX16 (human) siRNA-358	GGAUUAAGCAGAAGAUGAATT
4	Negative control	UUCUCCGAACGUGUCACGUTT
5	FAM-negative control	UUCUCCGAACGUGUCACGUTT
6	Positive control (human GAPDH)	GUAUGACAACAGCCUCAAGTT

### Transwell invasion assay

To evaluate the invasive ability of cells, the number of cells that migrate through the Matrigel-coated membrane was measured. Cell invasion was assessed using transwell chambers with an 8-µm pore membrane coated with 70 µL of Matrigel. Cells (5 × 10^4^ per well) were seeded in serum-free medium, and complete medium was added to the lower chamber. After 48 h, cells that invaded through the membrane were fixed, stained with crystal violet dye, and counted under a microscope. This assay provides a quantitative assessment of the cells’ migration and invasion potential.

### Wound healing assay

To assess cell migratory capacity, cells were seeded into 6-well plates, and a scratch was made in the cell monolayer using a 200-µL sterile pipette tip. All cells were cultured in serum-free medium. Images of the scratch area were captured at 0, 6, and 12 h for 786-O cells and at 0, 24 and 48 h for 769-P cells. The area of migration into the scratch was measured at each time point as an indicator of cell motility.

### CellTiter assay

Cell proliferation was quantified using the CellTiter-Glo^®^Luminescent Cell Viability Assay. After transfection, cells were plated into 96-well plates at a concentration of 4000 cells per well. Once the cells adhered, cell viability was assessed using the CellTiter-Glo^®^ Luminescent Assay Kit (Promega, USA) at time points of 0, 24, 48, 72, and 96 h. Luminescent signals (RLU) were detected using a microplate reader (TECAN Spark, Switzerland) and absorbance was measured at 562 nm. Comparative analysis was conducted between the NC and siRNA-treated groups to determine cell viability.

#### **Result**

##### Upregulated STX16 expression in ccRCC

**Fig. 2:**
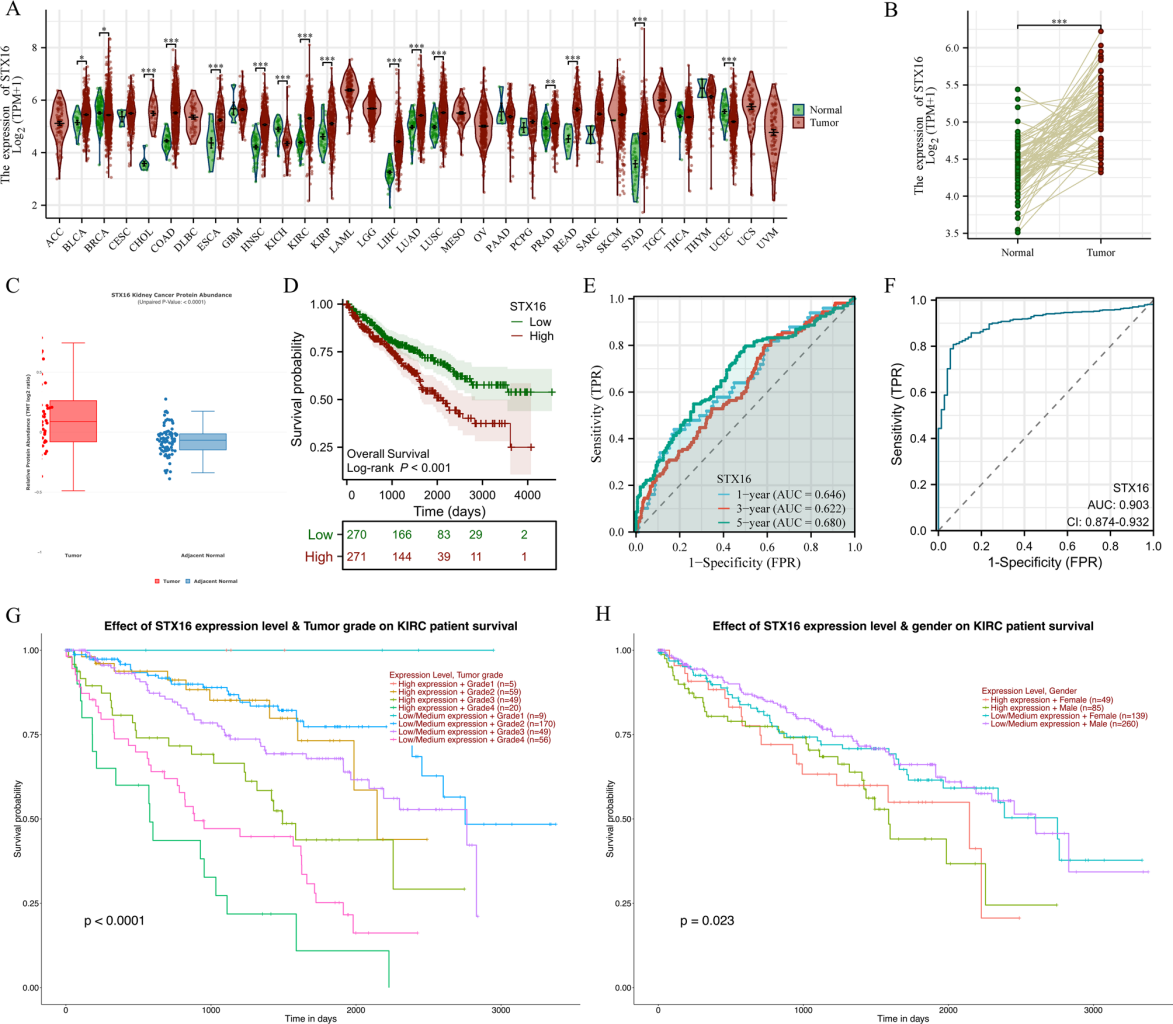
The expression levels of STX16 in ccRCC and its correlation with prognosis. (**A**) The mRNA expression levels of STX16 in pan-cancer analysis from TCGA dataset. (**B**) The STX16 mRNA expression of pairwise ccRCC samples in TCGA dataset. (**C**) The protein expression levels of STX16 in CTPAC dataset. (**D**) Kaplan–Meier survival curves indicated that ccRCC patients with high STX16 expression exhibited significantly worse OS than those with low expression. (**E**) ROC curve analysis predicted the accuracy of OS at 1, 3, and 5 years. (**F**) The diagnostic ROC analysis revealed a high diagnostic value of STX16. (**G**) The subgroup survival curves showed statistically significant differences across different histological grades. (**H**) The subgroup survival curves showed statistically significant differences between genders. *P < 0.05, **P < 0.01, ***P < 0.001; ccRCC, clear cell renal cell carcinoma; KIRC, kidney renal clear cell carcinoma; OS, overall survival; ROC, ‌receiver operating characteristic.

We analyzed the mRNA expression of STX16 using the TCGA database. The results showed that STX16 was significantly upregulated in 13 types of cancer tissues, compared to corresponding normal tissues (Fig. 2A). STX16 mRNA expression was significantly higher in kidney renal clear cell carcinoma (KIRC) compared with the normal tissues. The paired data, consisting of 613 ccRCC tissues and 72 corresponding adjacent normal kidney tissues, also revealed significantly higher STX16 expression in tumor tissues (*P* < 0.001, Fig. 2B). Additionally, data from CTPAC databases demonstrated increasing STX16 protein levels in ccRCC samples (Fig. 2C). These results conclusively indicated that STX16 was upregulated at both mRNA and protein levels in ccRCC.

### Correlation of STX16 expression with clinical and pathological features

**Fig. 3:**
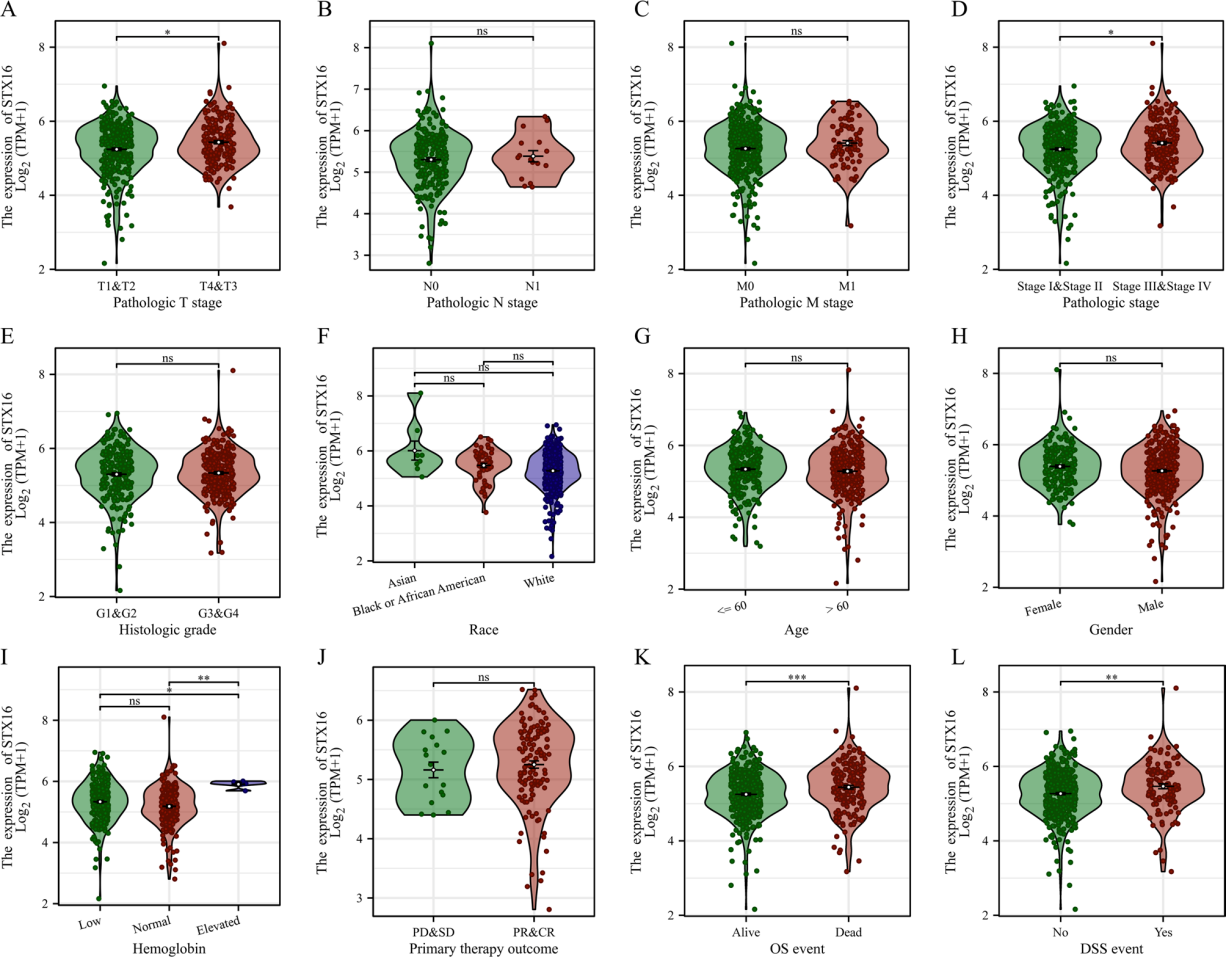
The correlation between the STX16 expression level and clinical and pathological characteristics of included patients. (**A**) Pathologic T stage; (**B**) Pathologic N stage; (**C**) Pathologic M stage; (**D**) Pathologic stage; (**E**) Histologic grade; (**F**) Race; (**G**) Age; (**H**) Gender; (**I**) Hemoglobin; (**J**) Primary therapy outcome; (**K**) OS event; (**L**) DSS event. *P < 0.05, **P < 0.01, ***P < 0.001, ns, no significance.

Based on the median STX16 expression level, the 613 ccRCC samples in the TCGA database were stratified into high and low expression groups. Figure 3A–L present the correlation between the STX16 expression level and clinical and pathological characteristics of all included patients. STX16 expression was significantly associated with T stage (*p* < 0.05), pathologic stage (*p* < 0.05), hemoglobin levels (*P* < 0.001), OS (*P* < 0.0001) and DSS (*P* < 0.001). Notably, STX16 expression progressively increased with advanced pathological stage. However, STX16 expression showed no significant correlation with N stage, M stage, histologic grade, race, age, gender, and primary therapy outcome.

### STX16 as a potential independent prognostic factor for ccRCC

To explore independent factors significantly associated with overall survival (OS) in ccRCC, univariable and multivariable Cox regression analyses were conducted, including STX16 expression and various clinical characteristics such as TNM stage, pathological stage, gender, race, age, histologic grade and serum calcium. The univariable analysis revealed that T stage (*P* < 0.001), N stage (*P* < 0.001), M stage (*P* < 0.001), pathological stage (*P* < 0.001), age (*P* < 0.0001), serum calcium (*P* < 0.001), and STX16 expression (*P* < 0.001) were significantly associated with OS. Multivariable analysis confirmed that age (*P* = 0.008) and STX16 expression (*P* < 0.0001) were independent prognostic factors for patients with ccRCC (Table 2).

**Table 2:** Univariable and multivariable Cox regression analyses of STX16 expression and clinicopathological characteristics of patients.

Characteristics	Total(*N*)	Univariate analysis		Multivariate analysis	
		Hazard ratio (95% CI)	*P* value	Hazard ratio (95% CI)	*P* value
Pathologic T stage	532				
T1	272	Reference		Reference	
T2	69	1.484 (0.891–2.471)	0.129	0.168 (0.015–1.878)	0.148
T3	180	3.249 (2.305–4.580)	**< 0.001**	0.583 (0.072–4.724)	0.613
T4	11	10.393 (5.253–20.562)	**< 0.001**	1.042 (0.088–12.290)	0.974
Pathologic N stage	256				
N0	240	Reference		Reference	
N1	16	3.395 (1.803–6.395)	**< 0.001**	0.344 (0.060–1.983)	0.232
Pathologic M stage	500				
M0	421	Reference		Reference	
M1	79	4.343 (3.184–5.924)	**< 0.001**	1.096 (0.091–13.155)	0.942
Pathologic stage	529				
Stage I	266	Reference		Reference	
Stage II	57	1.183 (0.638–2.193)	0.594	5.124 (0.372–70.659)	0.222
Stage III	123	2.592 (1.729–3.885)	**< 0.001**	3.101 (0.347–27.731)	0.311
Stage IV	83	6.478 (4.436–9.460)	**< 0.001**	12.713 (0.493–327.504)	0.125
Gender	532				
Female	187	Reference			
Male	345	0.944 (0.694–1.284)	0.714		
Race	525				
Asian	8	Reference			
White	461	1.886 (0.263–13.501)	0.527		
Black or African American	56	1.663 (0.214–12.894)	0.626		
Age	532				
<= 60	264	Reference		Reference	
> 60	268	1.779 (1.310–2.416)	**< 0.001**	2.088 (1.215–3.587)	**0.008**
Histologic grade	524				
G1	14	Reference		Reference	
G2	228	7537225.9701 (0.000–Inf)	0.993	4118507.2920 (0.000–Inf)	0.995
G3	206	13735528.0667 (0.000–Inf)	0.993	4012901.4205 (0.000–Inf)	0.995
G4	76	37109580.6512 (0.000–Inf)	0.993	8481476.2795 (0.000–Inf)	0.995
Serum calcium	364				
Low	204	Reference		Reference	
Normal	150	1.254 (0.885–1.776)	0.203	0.800 (0.476–1.345)	0.400
Elevated	10	4.846 (2.404–9.769)	**< 0.001**	0.892 (0.250–3.180)	0.860
STX16	532				
Low	266	Reference		Reference	
High	266	1.733 (1.280–2.345)	**< 0.001**	2.360 (1.419–3.926)	**< 0.001**

### High STX16 expression predicts poor prognosis

The prognostic significance of STX16 expression was evaluated by Kaplan-Meier survival analysis for OS in patients with ccRCC (Fig. 2D). Patients with high STX16 expression exhibited significantly worse OS than those with low expression (HR = 1.696 [95% CI 1.258–2.286], *P* < 0.001). The predictive accuracy of the survival model was demonstrated by ROC curves, with AUC values of 0.646, 0.622, and 0.680 at 1, 3, and 5 years, respectively (Fig. 2E). The diagnostic ROC analysis revealed that STX16 exhibited an AUC value of 0.903 in distinguishing ccRCC from healthy individuals, indicating a high diagnostic value (Fig. 2F). Subgroup survival analysis using the UALCAN platform revealed that STX16 expression levels (high vs. low) were associated with statistically significant differences in survival among patients with ccRCC. The subgroup survival curves showed statistically significant differences among histological grades (Fig. 2G; *p* < 0.001) and gender groups (Fig. 2H; *p* < 0.05). Subgroup analysis of OS further showed that high STX16 expression was associated with worse survival across different pathological stages. These results underscore the prognostic importance of high STX16 expression in ccRCC.

### Co-expression, DEGs and PPI network in ccRCC

**Fig. 4:**
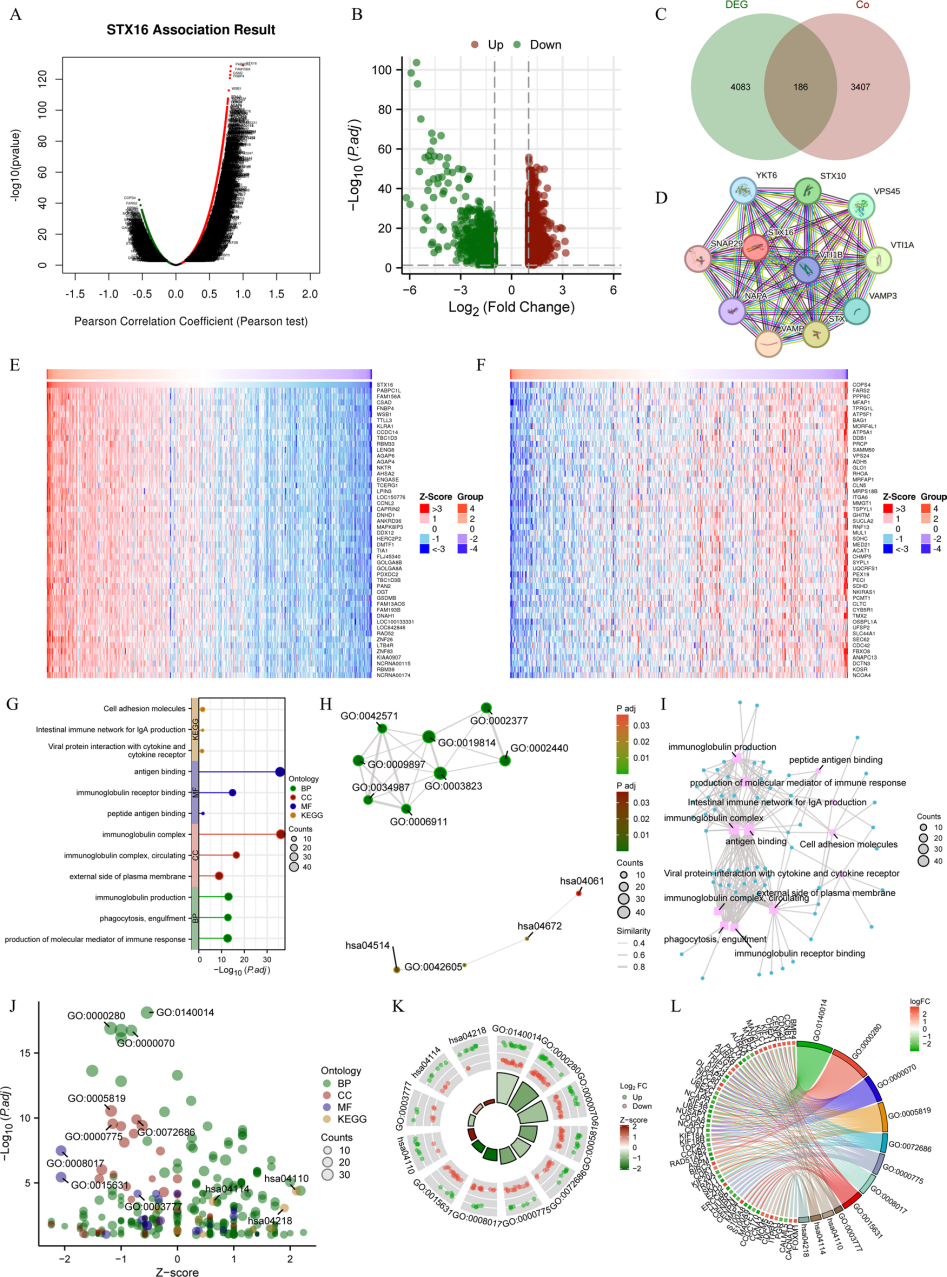
Co-expression, DEGs, PPI Networks, GO and KEGG analysis in ccRCC. (**A**) Volcano plots showing co-expression analysis of STX16. (**B**) Volcano plots depicting DEGs between high- and low-STX16 expression groups. (**C**) The Venn diagram presents 186 overlapping genes of co-expressed genes and DEGs. (**D**) PPI network for STX16. (**E**,**F**) Heatmaps displaying the co-expression patterns of the top 50 positively and 50 negatively correlated genes. (**G**–**I**) GO and KEGG analysis were visualized through lollipop plots, EMAP plots, and network diagrams. (**J**–**L**) GO and KEGG analysis integrated with logFC values were visualized through bubble plots, circle chart, and chord diagram. DEGs, differentially expressed genes; PPI, protein–protein interaction; ccRCC, clear cell renal cell carcinoma.

Co-expression analysis of STX16 was performed using Pearson test, and results was visualized through a volcano plot (Fig. 4A). Patients were divided into high- and low- expression groups based on the median expression level of STX16, and differentially expressed genes‌‌ (DEGs) between the two groups were analyzed and shown in Fig. 4B. A Venn diagram presents 186 overlapping genes between co-expressed genes and DEGs (Fig. 4C). Using the STRING database, a protein-protein interaction (PPI) network for STX16 was constructed to explore its potential functional partners in ccRCC. The network, as shown in Fig. 4D, revealed 10 key interacting proteins, including SNAP29, STX10, STX6, VAMP3, VAMP4, VPS45, VTI1A, VTI1B, and YKT6. The top 50 positively correlated genes and the top 50 negatively correlated genes were selected to construct co-expression heatmaps, revealing distinct expression patterns (Fig. 4E,F). These findings highlight the potential regulatory network and biological significance of STX16 in ccRCC.

### GO and KEGG analysis

GO and KEGG pathway analyses were conducted to investigate the functional roles of STX16 in ccRCC. Significant GO terms included “immunoglobulin production”, “immunoglobulin complex”, and “antigen binding” among others. KEGG pathway analysis identified associations with the “intestinal immune network for IgA production” and “cell adhesion molecules”. The results were visualized using lollipop plots (Fig. 4G), EMAP plots (Fig. 4H), and network diagrams (Fig. 4I). Additionally, GO and KEGG analysis integrated with logFC values provided a comprehensive overview of the biological processes and pathways linked to STX16 in ccRCC through bubble plots (Fig. 4J), a circle chart (Fig. 4K), and chord diagram (Fig. 4L).

### STX16 expression and immune infiltration in ccRCC

**Fig. 5:**
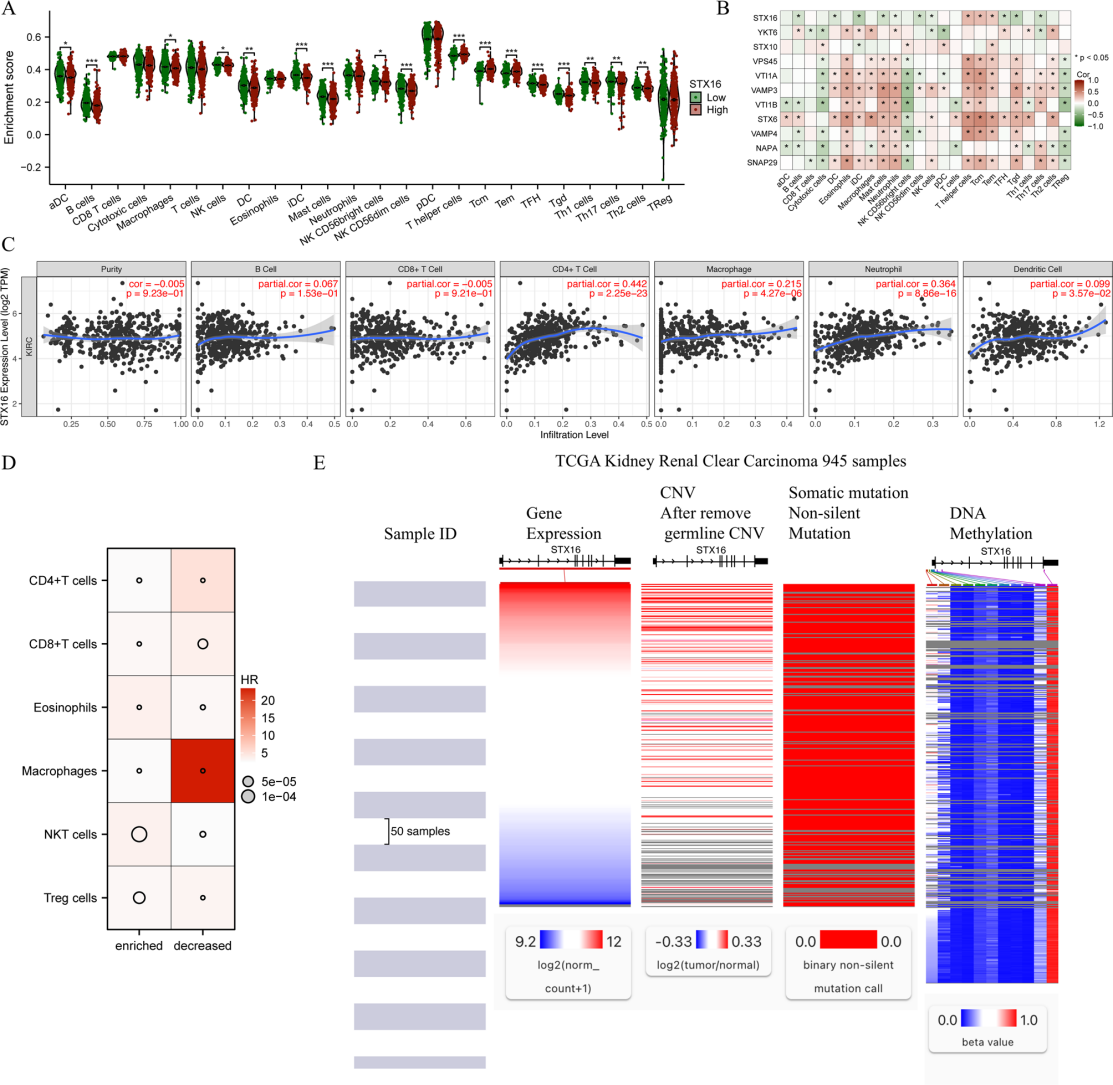
STX16 expression and immune infiltration in ccRCC. (**A**) Correlation between STX16 expression and immune infiltration in ccRCC. (**B**) Heatmap depicting the correlation between immune infiltration and STX16’s key interacting genes. (**C**) Correlation between STX16 expression and immune infiltration in ccRCC. (**D**) Heatmap depicting the prognostic impact of STX16 expression in ccRCC across cohorts with enriched and decreased infiltration of CD4 + T cells, CD8 + T cells, eosinophils, macrophages, NKT cells, and Treg cells. (**E**) Mutation, copy number variation, and DNA methylation analysis of STX16.

We assessed the relationship between STX16 expression and immune infiltration in ccRCC (Fig. 5A). STX16 expression was significantly correlated with central memory T cell, effector memory T Cell, and T helper cells (*P* < 0.001). We further visualized the relationship between immune infiltration and STX16’s key interacting genes using a heatmap (Fig. 5B). Using the TIMER database, we analyzed the correlation between STX16 expression and immune cell infiltration in ccRCC. STX16 expression was positively correlated with the infiltration levels of CD4 + T cells, macrophages, and neutrophils (Fig. 5C).

Patients were stratified into high- and low-STX16 expression groups based on the median expression level, and we investigated the Kaplan-Meier plotter database to assess the prognostic significance of STX16 expression in ccRCC across cohorts with enriched and reduced immune cell infiltration, which was integrated by heatmap in Fig. 5D. High STX16 expression was associated with a poor prognosis in KIRC across all cohort. Notably, in cohorts with enriched infiltration of CD4 + T cells, CD8 + T cells, macrophages, and Treg cells, the hazard ratios (HRs) were significantly lower than those in their respective reduced-infiltration. Conversely, in eosinophil- and NKT cell-enriched cohorts, the HR values were significantly higher compared their reduced-infiltration counterparts. These findings highlight the prognostic significance of STX16 expression in ccRCC and its differential impact depending on immune cell infiltration levels.

### Mutation, copy number variation, and DNA methylation analysis of STX16

To investigate the potential mechanisms underlying STX16 overexpression in ccRCC, we analyzed DNA methylation, gene mutations, and copy number variations (CNVs) using the UCSC Xena database, as these factors are critical regulators of genetic and epigenetic alterations in cancer. Heatmap analysis revealed the association between STX16 mRNA expression and CNV, DNA methylation, and somatic mutations in ccRCC (Fig. 5E).

### Single-cell analysis reveals intratumoral heterogeneity and tumor microenvironment

**Fig. 6:**
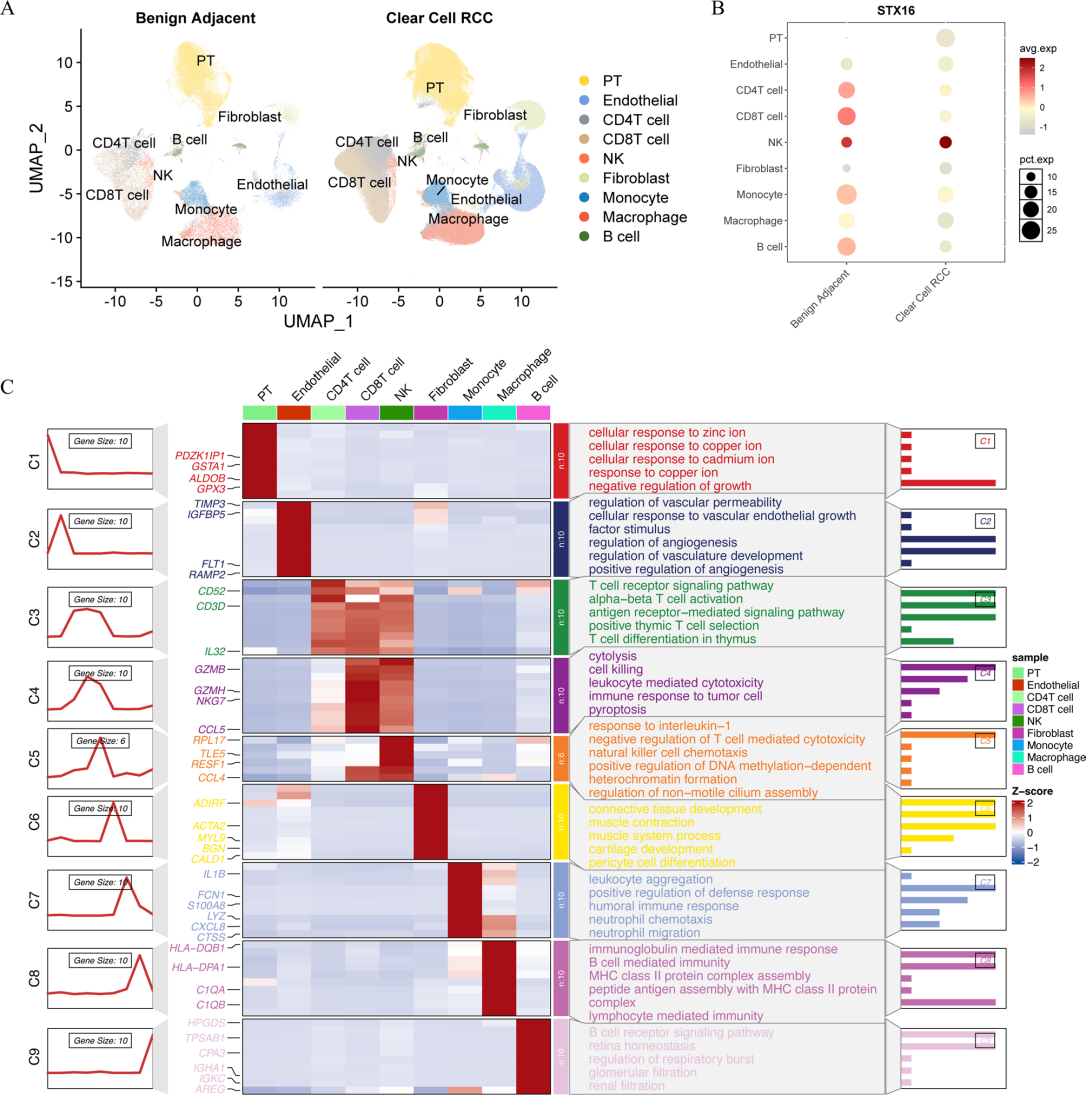
Single-cell analysis. (**A**) The UMAP plot illustrates the distribution of cell subclusters. (**B**) Differential expression of STX16 across distinct cell subclusters. (**C**) Cell populations were identified based on their characteristic genes.

Single-cell sequencing data from datasets GSE222703, GSE156632, GSE207493, GSE242299 and GSE159115 were downloaded, comprising 45 ccRCC samples and 25 adjacent healthy kidney tissues for analysis. The UMAP plot in Fig. 6A illustrates the distribution of cell subclusters, and cell populations were identified based on their marker genes as shown in Fig. 6C. Nine major cell types were identified, including PT cells, endothelial cells, CD4 + T cells, CD8 + T cells, NK cells, fibroblasts, monocytes, macrophages, and B cells. The differential expression of STX16 across distinct cell subclusters is shown in Fig. 6B.

### Validation of STX16 expression in ccRCC

**Fig. 7:**
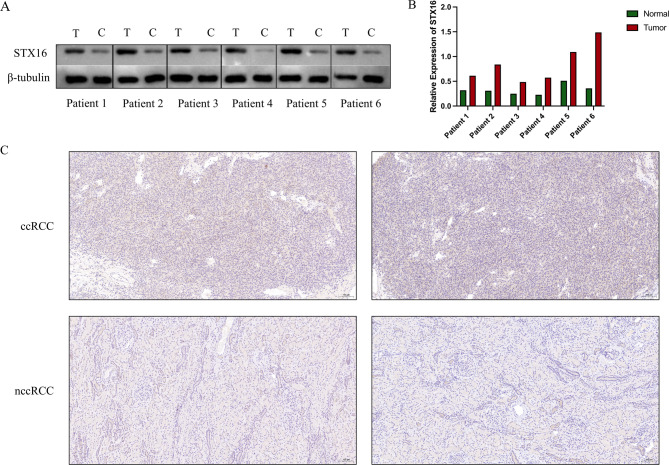
The protein expression levels of STX16 in ccRCC tissues. (**A**) Western blotting analysis demonstrated significantly higher STX16 expression levels in ccRCC tissues than in adjacent normal tissues. The grouping of blots was cropped from different parts of the same gel. (**B**) The column chart shows the grayscale analysis based on Western blotting. (**C**) Immunohistochemistry revealed stronger STX16 staining in ccRCC samples than in nearby normal kidney tissues.

Western blotting was performed on tissue samples from ccRCC patients, revealing significant differences in STX16 protein expression between tumor tissues and adjacent normal tissues (Fig. 7A) and the corresponding grayscale analysis is shown in the column chart (Fig. 7B). Protein expression levels of STX16 in ccRCC samples were further evaluated by immunohistochemistry (IHC), where tumor tissues displayed stronger STX16 staining compared to nearby normal kidney tissues (Fig. 7C).

### Validation of the relationship between STX16 expression and clinicopathological features in ccRCC in our cohort

In a cohort of 104 ccRCC patients from Beijing Tiantan Hospital, differential expression of STX16 was analyzed across subgroups based on gender, age, TNM classification, and histological grade (Table 3). Statistical analysis indicated that STX16 expression in ccRCC was correlated with tumor stage, T and N classifications (*p* < 0.05). No significant difference was detected for histological grade and M classification.

**Table 3:** The relationship between STX16 expression and clinicopathological features in CcRCC in our cohort.

Characteristics	Low expression	High expression	*P* value
n	52	52	
Age (years), median (IQR)	65.5 (60, 75)	68.5 (63.75, 74)	0.381
Histologic grade, n (%)			0.774
G1	3 (2.9%)	1 (1%)	
G2	17 (16.3%)	19 (18.3%)	
G3	23 (22.1%)	23 (22.1%)	
G4	9 (8.7%)	9 (8.7%)	
Stage, n (%)			0.022
I	36 (34.6%)	22 (21.2%)	
II	6 (5.8%)	7 (6.7%)	
III	4 (3.8%)	14 (13.5%)	
IV	6 (5.8%)	9 (8.7%)	
T classifcation, n (%)			0.023
T1	36 (34.6%)	24 (23.1%)	
T2	9 (8.7%)	8 (7.7%)	
T3	3 (2.9%)	13 (12.5%)	
T4	4 (3.8%)	7 (6.7%)	
N classifcation, n (%)			0.027
N0	43 (41.3%)	33 (31.7%)	
N1	9 (8.7%)	19 (18.3%)	
M classifcation, n (%)			0.485
M0	49 (47.1%)	46 (44.2%)	
M1	3 (2.9%)	6 (5.8%)	

### STX16 knockdown suppresses proliferation, migration, and invasion of 786-O and 769-P cells in vitro

**Fig. 8:**
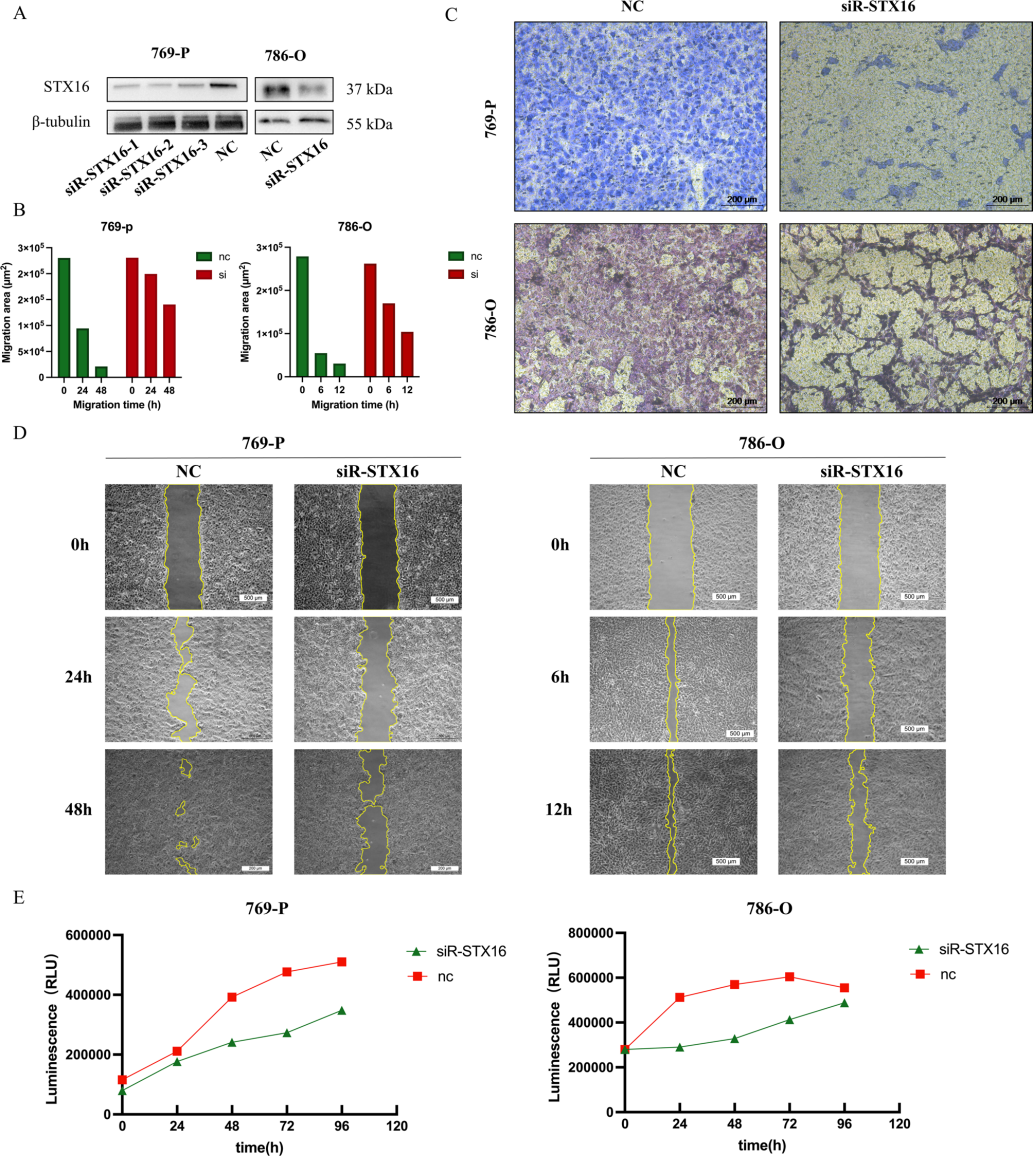
STX16 knockdown suppresses 769-P and 786-O proliferation, migration, and invasion in vitro. (**A**) Western blot analysis demonstrates a significant reduction in STX16 protein levels in 769-P and 786-O cell lines after STX16 knockdown. The grouping of blots was cropped from different parts of the same gel. (**C**) Transwell invasion assays showed a marked reduction in invasive capabilities in siR-STX16 cells. (**B**) Bar graphs were used to quantify the results of wound healing assays. (**D**) Wound healing assay indicated that knockdown of STX16 reduced the migratory ability of ccRCC cells. (**E**) Growth curves revealed that the knockdown of STX16 significantly inhibited the cell growth of both 769-P and 786-O cell lines.

To investigate the role of STX16 in ccRCC, STX16 siRNA and a scrambled siRNA negative control (NC) were transfected into 786-O and 769-P cell lines. The knockdown efficiency was confirmed using western blot analysis, which demonstrated a significant reduction in STX16 protein levels using each sequence of the 3 siRNAs (Fig. 8A). The impact of STX16 silencing on cell proliferation and invasion was subsequently assessed through various functional assays. Transwell invasion assays showed a marked reduction in invasive capabilities in siR-STX16 cells, as shown in Fig. 8C. Furthermore, wound healing assays was shown in Fig. 8D, and the results were quantified and presented as bar graphs, demonstrating the percentage of wound closure area. The data showed a significant reduction in the migration ability of 786-O and 769-P cells following STX16 knockdown (Fig. 8B). Cell viability was visualized by growth curves, which revealed that the knockdown of STX16 significantly inhibited the growth activity of both 786-O and 769-P cell lines (Fig. 8E).

## Discussion

As a member of the SNARE protein family, STX16 is involved in processes such as membrane fusion and intercellular communication, which are critical for cellular proliferation and migration and play a crucial role in cancer progression^17,18^. Prior research has predominantly examined STX16 in the context of metabolic diseases, leaving its involvement in cancer largely unexplored^19^. Our results represent the first detailed exploration of STX16 expression in ccRCC and are consistent with reports of altered STX16 levels in other malignancies, such as esophageal squamous cell carcinoma, suggesting STX16 might act as a shared oncogenic effector across epithelial malignancies^10^.

We demonstrated that STX16 is significantly overexpressed in ccRCC and independently associated with poor overall survival. Importantly, its expression correlates with advanced clinical parameters, including tumor stage and therapy outcomes. The role in prognosis and diagnosis positions STX16 as a critical molecular marker in the clinical management of ccRCC, highlights its potential as a clinical stratification tool. Importantly, the moderate yet consistent AUC values over time suggest a stable predictive ability, which is crucial for longitudinal patient monitoring.

The analysis of mutation, CNV, and methylation patterns of STX16 reveals potential mechanisms underlying its elevated expression in ccRCC. CNV analysis suggested that copy number gains might drive STX16 overexpression, a known mechanism for oncogene activation in cancer^20,21^. Additionally, reduced methylation levels in the STX16 promoter region may contribute to its transcriptional activation, which is a common epigenetic alteration in tumors^22^. Somatic mutations in STX16 were also detected, but their precise functional role in modulating expression and downstream oncogenic pathways remains unclear. Together, these genetic and epigenetic changes provide a multifaceted regulatory network that contributing to STX16 dysregulation in ccRCC, underscoring its potential as a therapeutic target for further investigation^22^.

The consistency of STX16’s prognostic impact across histologic grades and TNM stages emphasizes its central role in tumor evolution, regardless of differentiation status. Interestingly, the potential sex-specific survival differences observed warrant deeper investigation into whether STX16 expression is modulated by sex hormones or epigenetic regulators. Furthermore, the association of high STX16 expression with poorer survival across distinct pathological stages emphasizes its potential role in driving tumor progression. This strong performance highlights STX16 as a promising biomarker for ccRCC diagnosis. Its high sensitivity and specificity may support its integration into non-invasive diagnostic approaches, particularly in conjunction with liquid biopsy technologies. Future studies should validate these findings in larger, diverse cohorts to confirm their robustness across different populations. Clinically, these findings highlight the potential of STX16 as a diagnostic and prognostic biomarker, warranting further investigation into its role in tumor initiation and development.

Beyond clinical associations, our integrative analysis uncovered potential mechanistic underpinnings. Co-expression and PPI network analyses highlighted STX16’s interaction with multiple SNARE family members, suggesting that STX16 may act as a regulatory hub that integrates vesicular transport and immune signaling. The expression of STX16 may directly influence tumor progression, supported by STX16’s role in regulating membrane trafficking, which could facilitate the delivery of growth factor receptors and adhesion molecules to the plasma membrane, thereby enhancing proliferative and invasive capabilities. Mechanistically, STX16 may contribute to tumor growth by amplifying proliferative signaling or inhibiting apoptosis. For instance, interactions with syntaxins and vesicle-associated membrane proteins (VAMPs) may influence the secretion of pro-tumorigenic cytokines or immune checkpoints, thereby shaping the tumor microenvironment^23,24^. These findings highlight the necessity of exploring STX16-targeted interventions as part of personalized therapeutic strategies. Future studies should validate these findings in larger, multi-center cohorts and explore its therapeutic potential.

A central finding of this study is the immunological dimension of STX16. GO and KEGG enrichment analyses identified multiple immune-associated pathways such as antigen binding, immunoglobulin complex, immunoglobulin production, production of molecular mediators of the immune response and immunoglobulin receptor, reinforcing the hypothesis that STX16 may modulate the tumor immune microenvironment (TIME) by modulating the expression and activity of key immune-related genes and proteins. These interactions could impact the recruitment and activation of immune cells within the tumor microenvironment, thereby affecting the immune surveillance and immune evasion mechanisms of ccRCC^25–27^. Understanding how STX16 regulates such processes could provide critical insight into ccRCC’s immune-evasive mechanisms and identify potential vulnerabilities for combination immunotherapy.

We also explored the relationship between STX16 expression and immune cell infiltration in ccRCC. Our results demonstrated a significant positive correlation between STX16 expression and the infiltration of immune cells, suggesting that STX16 may play an important role in enhancing adaptive immune responses in the tumor microenvironment. These T cell subsets are essential for immune surveillance and the maintenance of anti-tumor immunity, which reflects that STX16 may contribute to the activation and persistence of tumor-specific T cell responses^28^. Additionally, we found a positive correlation between STX16 expression and the infiltration level of CD4 + T cells, macrophages and neutrophils. CD4 + T cells are critical for orchestrating immune responses, while macrophages and neutrophils, depending on their polarization, can either support anti-tumor immunity or facilitate tumor progression^29^. The positive association suggests that STX16 may modulate the recruitment and activation of these immune cells to promote an immune response against the tumor. However, STX16 also showed a negative correlation with other immune cell types, which may reflect its selective influence on immune cell dynamics and its potential role in immune escape mechanisms.

Elevated STX16 expression was consistently associated with poor prognosis, regardless of immune cell infiltration levels, highlighting its potential role as a driver of tumor progression and a therapeutic target. Immune populations may exert a protective anti-tumor effect, counteracting the negative impact of high STX16 levels. Furthermore, current advancements in immunotherapy, particularly immune checkpoint inhibitors targeting PD-1/PD-L1 or CTLA-4, have demonstrated significant efficacy in ccRCC^30–32^. Combining STX16-targeted therapies with existing immunotherapeutic approaches could synergistically enhance anti-tumor immune responses by reducing immune evasion associated with STX16 expression and improving the overall immune landscape within the tumor microenvironment. However, the precise mechanisms by which STX16 modulates immune cell function and contributes to tumor progression are yet to be elucidated and warrant comprehensive experimental validation.

Single-cell transcriptomic analysis provided a high-resolution map of STX16 expression across diverse cell types in ccRCC and adjacent tissues, highlighting its enrichment not only in tumor epithelial cells but also in immune and stromal compartments. The TIME is a critical determinant of ccRCC progression and therapeutic response^33^. This spatial heterogeneity suggests context-specific roles for STX16 that could vary between immunostimulatory and immunosuppressive microenvironments. Recognizing the impact of technical variability, our integrative approach mitigates dataset bias and provides a more reliable picture of STX16’s role in the TIME. Future spatial proteomics and single-cell CRISPR screens could further clarify these roles. Notably, the detection of STX16 expression in key immune and stromal cell populations underscores its potential as a significant regulator of the TIME in ccRCC. Future investigations should focus on unraveling the biological drivers of this heterogeneity and assessing its clinical relevance, particularly in the context of using STX16 as a biomarker or therapeutic target^34^.

Moreover, STX16’s interaction with other SNARE proteins might influence immune signaling by modulating cytokine secretion or immune checkpoint expression. Such a role positions STX16 not only as a tumor-intrinsic factor but also as a regulator of tumor–immune interactions, potentially affecting the efficacy of immunotherapies. Our data thus support the rationale for combining STX16-targeted approaches with immune checkpoint inhibitors to enhance therapeutic response.

Western blot and IHC analyses confirmed that STX16 was overexpressed in ccRCC tumor tissues relative to normal tissues, consistent with bioinformatics results. Clinically, higher STX16 expression correlated with advanced tumor stage and higher T and N classification, supporting its role in tumor growth and lymph node metastasis. Functional assays revealed that STX16 knockdown significantly inhibited ccRCC cell proliferation, migration, and invasion. Reduced cell viability and impaired invasive capabilities further highlight STX16’s role in promoting tumor aggressiveness. As a potential prognostic biomarker and therapeutic target, further research should explore its molecular mechanisms and interactions within the tumor microenvironment.

Lysosomes are integral to maintaining cellular homeostasis by degrading and recycling intracellular components^35^. STX16, as a key component of the SNARE protein family, modulates the fusion of autophagosomes with lysosomes, a key step in the autophagic pathway^17^. This not only supports the degradation of damaged organelles and misfolded proteins but also facilitates the recycling of essential nutrients and macromolecules^36^. Beyond its role in vesicle fusion, STX16 appears to intersect with autophagy and lysosomal degradation—pathways essential for tumor cell survival under stress. Cancer cells exploit autophagy to withstand metabolic and therapeutic challenges, and STX16-mediated lysosomal fusion may be pivotal in this adaptation^37^. A disruption in STX16-mediated membrane fusion may impair lysosomal degradation, leading to the accumulation of cellular waste and promoting the progression of cancer by increasing cellular stress and inducing inflammatory responses. Inhibiting STX16 in certain cancers may disrupt this survival pathway, making tumor cells more susceptible to therapies that target autophagy^38^. Notably, by affecting the trafficking and fusion of lysosomes within immune cells, STX16 may influence the activation of immune responses and potentially contribute to immune evasion in tumors^39^. Disruption of STX16 may, therefore, alter immune cell behavior, impairing the tumor’s ability to mount an effective immune response. Given STX16’s central role in regulating lysosomal function, autophagy, and immune surveillance, targeting STX16 represents a promising therapeutic strategy in cancer treatment.

Despite the comprehensive nature of this study, certain limitations should be acknowledged. The clinical sample size was relatively small and sourced from a single center, potentially introducing selection bias. Multicenter studies with larger cohorts are needed to confirm the generalizability of these findings. Additionally, while this study provides strong evidence for STX16’s functional role in vitro, in vivo studies, including animal models, are necessary to validate its biological relevance. Finally, the translational potential of STX16 as a th’rapeutic target remains to be fully explored. Future research should evaluate its therapeutic potential in combination with existing treatments, such as immune checkpoint inhibitors, to enhance therapeutic outcomes.

## Conclusion

This study provides the first comprehensive analysis of STX16 in ccRCC, elucidating its expression pattern, clinical significance, and potential mechanisms of action. These findings highlight STX16 as a promising diagnostic and prognostic biomarker, as well as a potential therapeutic target, paving the way for personalized treatment strategies in ccRCC.

## Supplementary Information

Below is the link to the electronic supplementary material.

Supplementary Material 1

## Data Availability

The datasets analysed during the current study are available in the TCGA repository, [https://portal.gdc.cancer.gov/]. The datasets analysed during single-cell sequencing analysis are available in the GEO repository, [https://www.ncbi.nlm.nih.gov/gds]. The accession number is GSE222703, GSE156632, GSE207493, GSE242299 and GSE159115. All original contributions from this study are included in the main article and supplementary materials.For additional information or inquiries, please contact the corresponding authors.

## Abbreviations

AUC: Area under the curve
BP: Biological process
ccRCC: Clear cell renal cell carcinoma
CC: Cellular component
CNV: Copy number variation
CTPAC: Clinical proteomic tumor analysis consortium
DEGs: Differentially expressed genes
DMEM: Dulbecco’s modified eagle medium
DSS: Disease-specific survival
FBS: Fetal bovine serum
GEO: Gene expression omnibus
GO: Gene ontology
GSVA: Gene set variation analysis
HR: Hazard ratio
IHC: Immunohistochemistry
KIRC: Kidney renal clear cell carcinoma
MF: Molecular function
NC: Negative control
OS: Overall survival
PPI: Protein–protein interaction
qRT-PCR: Quantitative reverse transcription polymerase chain reaction
ROC: Receiver operating characteristic
scRNA-seq: Single-cell RNA sequencing
siRNA: Small interfering RNA
SNARE: Soluble *N*-ethylmaleimide-sensitive factor attachment protein receptor
ssGSEA: Single-sample gene set enrichment analysis
STX16: Syntaxin 16
TCGA: The cancer genome atlas
TIMER: Tumor immune estimation resource
TNM: Tumor, node, metastasis
UALCAN: University of Alabama at Birmingham Cancer Data Analysis Portal
WB: Western blot
