# Supplementary material for: Integrative analysis identifies STX16 as a prognostic and immune-associated biomarker in ccRCC
Source: Sci Rep. 2025 Nov 20;15:41094. doi: 10.1038/s41598-025-24921-9 (PMC12634691; doi:10.1038/s41598-025-24921-9)
Supplement: Supplementary file 1 — Supplementary Material 1 [file 41598_2025_24921_MOESM1_ESM.docx]

**Supplementary Legend**

Figure S1. The efficiency of the three designed primers was validated using qRT-PCR.

Figure S2. Western blot analysis in STX16 protein levels in ccRCC and normal tissues.

Figure S3. Western blot analysis in STX16 protein levels in 769-P cell lines after STX16 knockdown.

Figure S4. Western blot analysis in STX16 protein levels in 786-O cell lines after STX16 knockdown.
